# Empowering Capacitive Devices: Harnessing Transfer Learning for Enhanced Data-Driven Optimization

**DOI:** 10.1021/acs.iecr.4c01171

**Published:** 2024-06-29

**Authors:** Teslim Olayiwola, Revati Kumar, Jose A. Romagnoli

**Affiliations:** † Cain Department of Chemical Engineering, Louisiana State University, Baton Rouge, Louisiana 70803, United States; ‡ Department of Chemistry, Louisiana State University, Baton Rouge, Louisiana 70803, United States

## Abstract

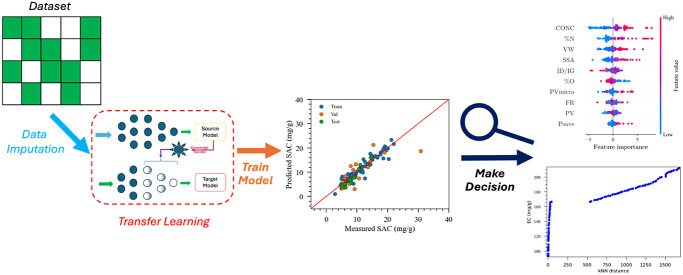

Developing data-driven models has found successful applications in engineering tasks, such as material design, process modeling, and process monitoring. In capacitive devices like deionization and supercapacitors, there exists potential for applying this data-driven machine learning (ML) model in optimizing its potential use in energy-efficient separations or energy generation. However, these models are faced with limited datasets, and even in large quantities, the datasets are incomplete, limiting their potential use for successful data-driven modeling. Here, the success of transfer learning in resolving the challenges with limited datasets was exploited. A two-step data-driven ML modeling framework named *ImputeNet* involving training with ML-imputed datasets and then with clean datasets was explored. Through data imputation and transfer learning, it is possible to develop a data-driven model with acceptable metrics mirroring experimental measurements. By using the model, optimization studies using the genetic algorithm were implemented to analyze the solution under the Pareto optimality. This early insight can be used in the initial stage of experimental measurements to rapidly identify experimental conditions worthy of further investigation. Moreover, we expect that the insights from these results will drive accurate predictive modeling in other fields including healthcare, genomic data analysis, and environmental monitoring with incomplete datasets.

## 1 Introduction

Reducing carbon emissions is a critical concern in the scientific community, especially within the chemical industry,^1−3^ as scientists tackle the challenge of minimizing emissions from industrial processes such as energy generation^4,5^ and water desalination.^6−8^ In the pursuit of developing energy-efficient devices with high separation efficiency, researchers are exploring modifications in materials and process variables for capacitive devices such as supercapacitors (SCs) and capacitive deionization (CDI) devices. CDI, in particular, has gained prominence as a preferred method for water desalination due to its energy efficiency, environmental friendliness, and operational simplicity.^9,10^ It works by the electrosorption of ions onto porous electrode surfaces through the application of an electric potential, enabling ion storage and absorption within the electrode material.^11−13^ Key components of CDI include carbonaceous cathode and anode electrodes, a feed stream, and an applied voltage.^11−13^ Similarly, supercapacitors, also known as ultracapacitors or electric double-layer capacitors, store energy via electrostatic charge separation and are employed in high-power applications like regenerative braking, offering higher power density but lower energy density than batteries.^14,15^ Despite their different applications—with CDI for water desalination and supercapacitors for high-power energy storage—both devices share the principles of an electrical double layer (EDL) upon application of electric potential and components like carbonaceous electrodes.^14,16−18^ Efforts to optimize these devices have been focused on enhancing the supercapacitor capacitance and CDI salt adsorption capacity through experimental and theoretical research. Notably, the activated carbon’s high specific surface area, electrical conductivity, and tunable pore structure have significantly improved CDI’s salt adsorption efficiency,^19,20^ while features such as doping with heteroatoms have markedly increased the supercapacitor capacitance depending on the material type.^21−23^ These findings underscore the importance of investigating factors such as the specific surface area, dopant concentration, current density (or voltage), and electrolyte concentration to further enhance the device performance.

In the quest to optimize the capacitance (CAP) of supercapacitors (SCs) or the salt adsorption capacity (SAC) of CDI, machine learning (ML) techniques can be instrumental in determining the ideal range of attributes. However, ML models often require large datasets, and researchers frequently face the challenge of limited or incomplete data, which hampers the development of successful data-driven models. To address this issue, researchers have employed strategies, such as imputation with ML or data augmentation. For instance, Deshsorn et al.^23^ used KNN imputation on a dataset of 513 records, including specific surface areas, defect ratios, and dopant percentages, to enhance their stacked ML model, achieving an absolute mean error of 14.3 F/g. Likewise, Saffarimiandoab et al.^19^ developed an ML model to predict the SAC of CDI with an RMSE of 2.271 mg/g using an imputed dataset featuring variables like applied voltage, electrolyte concentration, and electrode pore characteristics. Data augmentation, particularly useful for small datasets, has been successfully applied by researchers like Adeleke et al.,^24^ Han et al.,^25^ Okolie et al.,^26,27^ For example, Okolie et al.^26^ improved the predictive performance of a multioutput ML model for lignocellulosic biomass composition from a correlation coefficient of 0.7233 to 0.854 by adding data generated from the generative adversarial network (GAN) model proposed by Goodfellow et al.^28^ Also, Han et al.^25^ reported an increase in predictive accuracy for protein solubility from 0.4258 to 0.4383 using a GAN-augmented ML model. These efforts highlight the significance of data augmentation in enhancing the ML model performance. Nonetheless, the potential of augmented data to disrupt the correlation within the original dataset poses a risk, and both imputation and augmentation methods could lead to inaccurate models if not carefully implemented.

To resolve the possible anomaly that might arise in the combination of actual and synthetic datasets, the success of the transfer learning (TL) protocol could be exploited. In TL training, given a source domain *D*_s_ and learning task *T*_s_, and a target domain *D*_T_ and a learning task *T*_T_, TL aims to help the learning of the target predictive function *f*_T_(·) for the target domain using the knowledge in *D*_s_ and *T*_s_, where *D*_s_ ≠ *D*_T_ and *T*s ≠ *T*_T_.^29^ The remarkable success of TL has been shown in fields such as materials informatics,^30−34^ process modeling,^35−37^ and process monitoring.^38−41^ In these works, researchers have used different sets of data types including simulated data from empirical or process simulation,^29,41^ experimental data from related studies, and fake data from generative models^39^ to pretrain the ML model before fine-tuning the target problem. Generally, these works showed the positive application of how to use TL to address data limitations affecting the development of accurate data-driven modeling.

In this work, a TL approach is presented for modeling supercapacitors and capacitive deionization by utilizing an imputed dataset (obtained from imputation of rows with incomplete columns) as the source domain and a complete experimental dataset (excluding rows with incomplete columns) as the target domain. Herein, we leveraged the dataset from Deshsorn et al.^23^ (out of 620 rows, only 159 complete rows) and Saffarimiandoab et al.^19^ (583 data points with only 105 complete rows) relating to supercapacitors and capacitive deionization, respectively. The first aspect addressed is the predictability and potential errors arising from the imputation of missing rows. Subsequently, a TL strategy to resolve anomalies from a large amount of missing data in data-driven modeling is discussed. Furthermore, the impact of the available features on the studied outputs, specifically the salt adsorption capacity and specific capacitance, is extracted from the trained TL models. Finally, the implementation of metaheuristic-based optimization in determining the optimized conditions of the two devices is demonstrated. This approach aims to enhance the modeling of supercapacitors and capacitive deionization devices by effectively utilizing imputed and experimental data through transfer learning, while also exploring the influence of features on the studied outputs and optimizing device conditions.

## 2 Implemented Methodology

**Figure 1:**
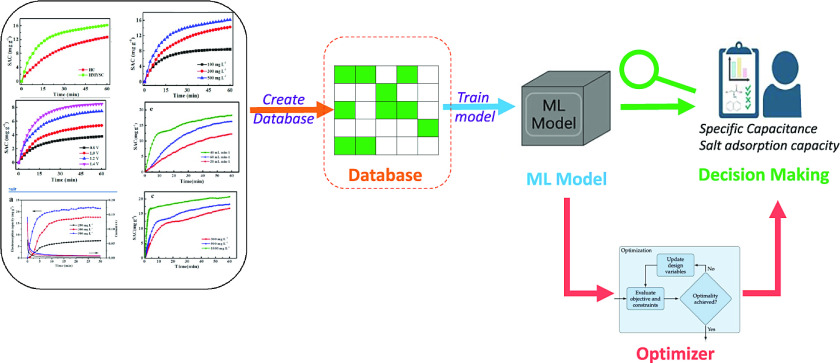
The framework “*ImputeNet”* employed in this study showing the database creation, machine learning model, and optimization framework.

The comprehensive strategy of this study is elucidated in Figure 1. Initially, an imputation-based model for capacitive devices served as the source domain, with complete yet constrained experimental datasets utilized as target domains. In the ML model, a fully connected neural network, optimized through a Bayesian optimization algorithm-based approach, was implemented. Subsequently, a meticulous examination of the significance of various parameters on the model outputs was conducted. Following this analysis, an optimization process was undertaken to ascertain the optimal set of parameters, enhancing the capacity to design novel materials and devices more effectively.

### 2.1 Description of Available Data

**Figure 2:**
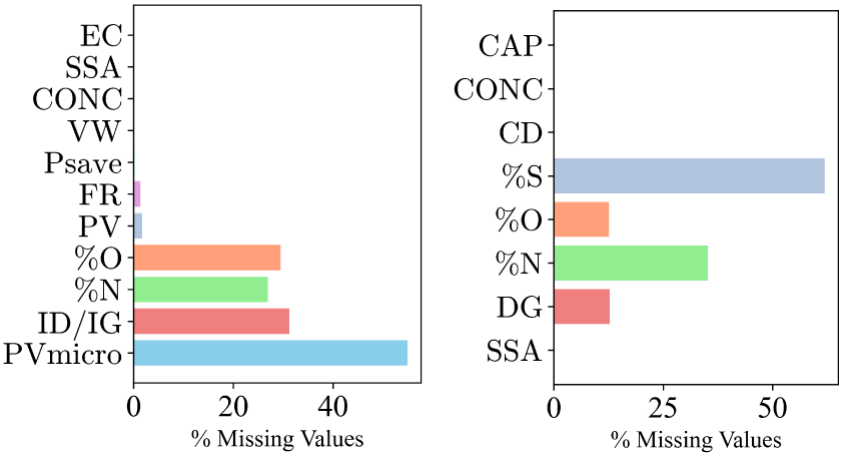
Number of missing values for cases D1 (left) and D2 (right). The total number of data points for cases D1 and D2 are 583 and 620, respectively.

In this study, we utilized datasets pertaining to capacitive devices. Specifically, we sourced the data from the works of Deshsorn et al.^23^ and Saffarimiandoab et al.^19^ Firstly, case D1 authored by Saffarimiandoab et al.^19^ reported the information on salt adsorption capacity or electrosorption capacity of capacitive deionization systems under varying conditions of applied voltage window (VW), electrolyte (NaCl) concentration (CONC), average electrode pore size (PSave), electrode pore volume (PV), electrode micropore volume (PVmicro), stream flow rate (FR), specific surface area (SSA), percentage of nitrogen dopant (%N), oxygen dopant (%O), and sulfur dopant (%S). On the other hand, case D2, as reported by Deshsorn et al.,^23^ provided the specific capacitance (CAP) of supercapacitors. This dataset includes features such as specific surface area (SSA), defect ratio (DG), percentage of nitrogen dopant (%N), oxygen dopant (%O), and sulfur dopant (%S), current density (CD), and electrolyte concentration (CONC). Both datasets exhibit instances of missing data, and the corresponding percentages of which are depicted in Figure 2. The distribution of the two datasets is listed in Tables 1 and S1 with case D1 initially comprising 583 data points; however, after removing the rows with missing data, 105 points remain. Similarly, case D2 contained 620 rows, with 159 rows remaining after dropping those with missing information.

**Table 1:** Summary of Statistical Analysis of Case D1.

	VW	FR	CONC	SSA	PV	Psave	PVmicro	ID/IG	%N	%O	EC
count	583	575	583	583	573	581	263	401	426	411	583
mean	1.26	28.27	386.29	1119.61	0.95	4.35	0.35	1.03	3.41	8.16	10.49
std	0.30	17.79	380.78	773.89	0.66	4.17	0.27	0.19	3.71	4.71	7.10
min	0.4	2.5	20	4.5	0.02	0	0	0.52	0	0	0.05
25%	1.2	15	100	586.9	0.5	2.07	0.13	0.91	0	5.3	5.51
50%	1.2	25	292.5	907	0.78	2.89	0.27	1.02	2.67	7.7	9.7
75%	1.4	40	500	1450.6	1.25	4.87	0.5	1.1	4.9	10.24	14.18
max	2	100	5000	4482	4.2	23.71	1.06	1.62	14.33	32.09	65

### 2.2 Imputation of Missing Data

In the domain of data imputation, we studied two robust imputation methodologies: the *k*-nearest neighbors (KNN) and the multivariate imputation by chained eq (MICE) algorithms. Within the MICE framework, the imputation of missing values unfolds through an iterative process driven by predictive models.^42^ In each iteration, the missing values of a specific variable are predicted by leveraging the information from other variables present in the dataset. On the contrary, the KNN imputation strategy involves the meticulous completion of missing values by assessing the nearest neighbors.^43^ For each sample, the missing values are imputed using the mean value derived from a predefined number of neighboring samples identified within the training set. The closeness between the two samples is ascertained through the similarity of features for which neither samples exhibit missing data.

In the MICE algorithm, we opted for a nonlinear model, specifically the extremely randomized trees (ETR),^44^ to serve as the predictive model. This decision was motivated by the desire to capture intricate relationships within the dataset for robust imputation outcomes and the successful application in the works of Yuan et al.^45^ To assess and compare the predictive efficacy of these imputation models in reconstructing missing data within a dataset, we introduced artificial missing data into an initially complete dataset, devoid of any missing values. For example, in case D1, we have only 105 rows with complete columns out of a total of 583 samples, and we randomly delete rows in a specific column (namely PVmicro, EC, and FR) to create 20%, 50%, and 70% incomplete data. Furthermore, our investigation extends beyond mere imputation accuracy to scrutinize the imputation algorithm’s capability to faithfully reproduce experimental values under varying conditions. This comprehensive analysis involves systematically introducing an escalating percentage of missing values into the dataset, allowing us to gauge the resilience and reliability of the imputation methodology across a spectrum of data completeness scenarios.

### 2.3 Training the Data-Driven Model via Transfer Learning

**Figure 3:**
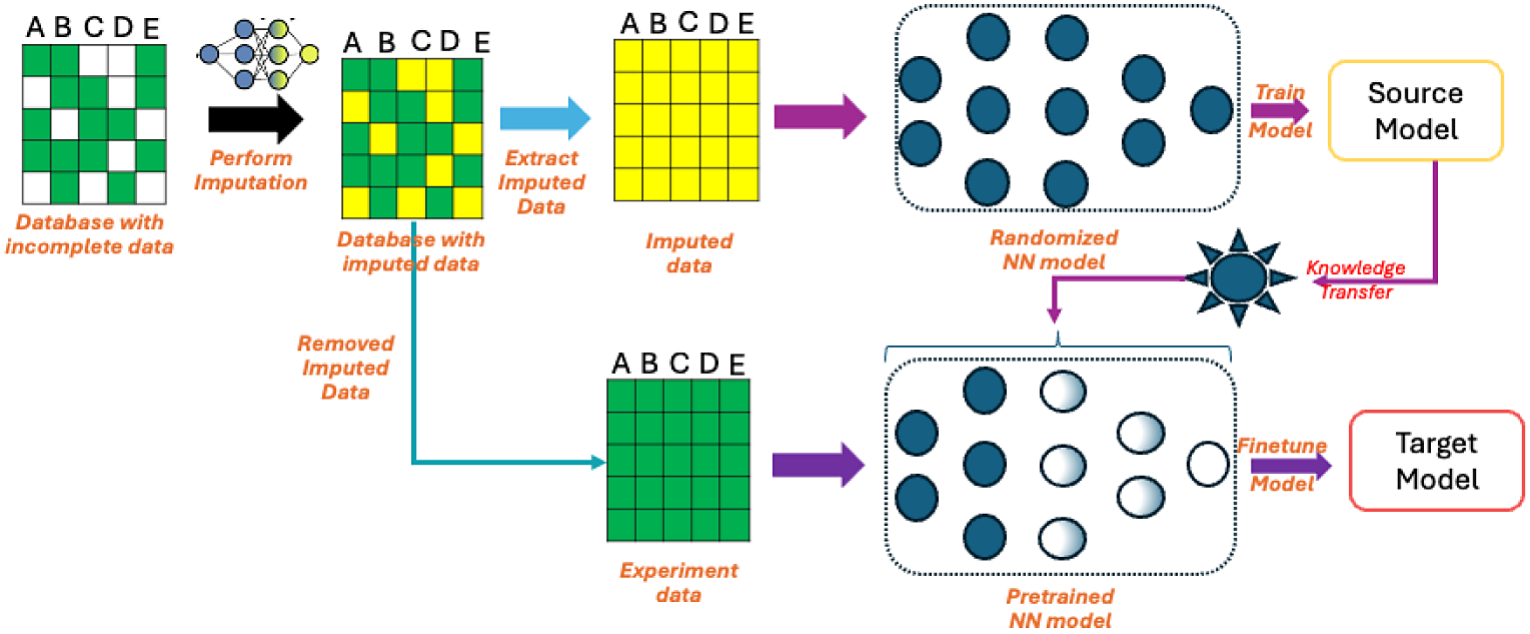
The framework showing the methodical approach employed in handing incomplete rows/columns, pretraining, and fine-tuning the ML model. The white , green , and yellow spaces represent the missing information, experimentally determined value, and ML-imputed value, respectively.

For model development, the TL protocol shown in Figure 3 was harnessed to enhance the learning of a target predictive function. Herein, a neural network (NN), specifically the fully connected NN, was adopted in this study due to its ability to extract the contribution of different layers (defined by the model weights and biases) and inputs to the output. In the TL process, two sets of data, namely (1) imputed data generated from the imputation technique (i.e., rows with incomplete columns), corresponding to 461 and 478 for cases D1 and D2, respectively and (2) experimental data (i.e., rows with complete columns) corresponding to 105 and 159 samples for D1 and D2, respectively, were obtained by dropping the rows of the imputed dataset from the whole data.

In training the source model *f*_T_(·) based on *D*_s_, the imputed dataset was used as the sample set. In training the source model, the Bayesian optimization, facilitated by the KerasTuner^46^ optimizer based on Keras—a Python-based deep learning API running on TensorFlow^47^—was employed to optimize parameters such as the number of layers (1–5), neurons per layer (1–50), and learning rate (10^–2^, 10^–3^, 10^–4^, and 10^–5^). The Bayesian optimizer minimizes the mean squared error of validation, utilizing a maximum of 100 trials, seed 42, and an early stopping criterion with a patience of 40. Following the identification of optimized hyperparameters, they were employed to train a source model using the same training and validation sets. Post-source model development, the model weights were preserved and then transferred to a new model, which underwent fine-tuning using the clean dataset. We fine-tuned through the full network based on the fact there exists a large new dataset similar to the source model dataset.

In the ML framework, the available datasets, comprising either imputed or experimental data, were partitioned into training, validation, and testing sets, adopting an 80:15:5 ratio, respectively. During training, the model exclusively encountered the training and validation sets. The validation set served to assess the model’s performance in predicting salt adsorption capacity (EC) or specific capacitance (CAP) on data not included in the training set, providing insights into the model’s ability to generalize and accurately predict unseen data.

Understanding the learning protocols of the ML model for the capacitive device design can be intricate. To elucidate the contributions of different feature parameters to predicted outputs, the SHAP (SHapley Additive exPlanations) framework by Lundberg and Lee^48^ was employed. SHAP assigns a Shapley value to each feature, representing its contribution to the model output. The Shapley values are visualized through bar plots or waterfall plots, arranged in the descending order of importance in the former and showing positive or negative contributions in the latter. Shapley values were calculated utilizing the DeepExplainer, an enhanced version of the DeepLIFT algorithm tailored to approximate SHAP values for deep learning models.^48^ This comprehensive approach ensures a thorough exploration of the model performance and interpretability in the context of capacitive device design.

### 2.4 Property Optimization

The primary objective of predictive models is to enhance the development of superior designs. Figure S5 shows the multi-objective optimization framework employed in this study. This objective is manifested in the exploration of the input space of models to identify combinations that result in favorable performance metrics. The problem at hand can be cast as a multi-objective optimization task, as shown in eq 1. In this equation, ‘*f*’ denotes the EC for case D1 or CAP for case D2 estimated through a surrogate model. Meanwhile, ‘dist’ represents the KNN distance between a candidate solution and the samples in the training space. The variables ‘*l*_*i*_’ and ‘*u*_*i*_’ signify the lower and upper bounds of each decision variable (inputs in the ML model), respectively, set based on the experimental data. Additionally, it is experimentally known that CAP/EC is non-negative. By maximizing CAP or EC while minimizing the KNN distance, the optimization process is steered away from potentially unreliable solutions (POEs) toward more dependable outcomes. The optimization problem outlined in eq 1 was successfully addressed using NSGA-II, employing a population size of 100 and 50 offspring, and spanning 50 generations.
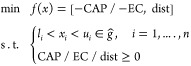
1

## 3 Results and Discussion

In the following section, the results of the comparison of the ability of two common imputation approaches to reproduce the experimental data, transfer learning to resolve the possible abnormality in the dataset arising from imputation, inferred knowledge learning protocol of the developed model, and finally, the optimized conditions of the features are presented in this section.

### 3.1 Verification of the Imputation Methods

**Figure 4:**
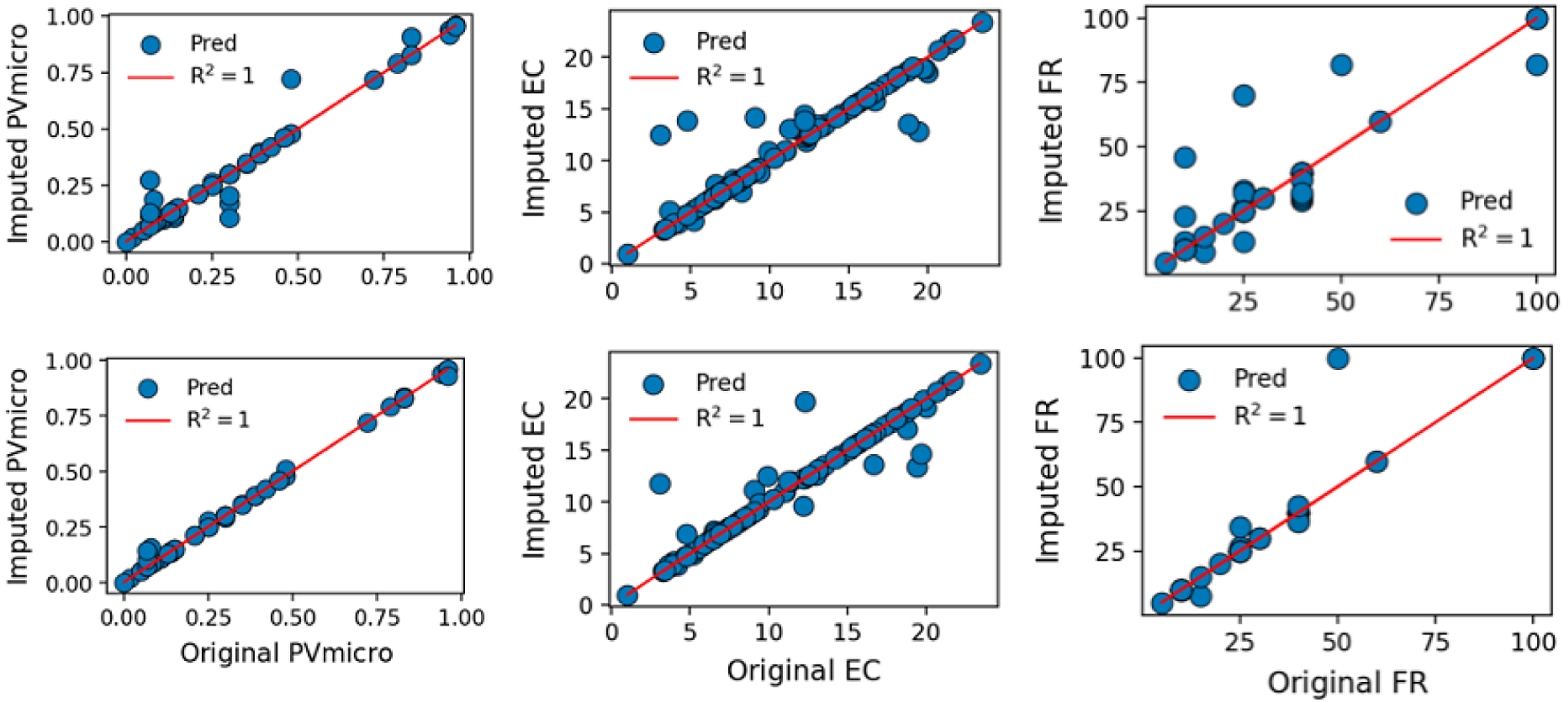
Comparison of two data imputation methods for case D1 (salt adsorption capacity, mg/g). Top (KNN) and bottom (ETR).

To assess the effectiveness of different imputation methods, specifically KNN and MICE-based ETR, we conducted imputations on a dataset with artificially induced missing data. The comparative analysis, illustrated in Figure 4, focused on 20% missing data for properties such as PVmicro, EC, and FR from case D1. Also, Figure S1 shows a similar analysis for case D2 variables namely %N, SA, and DG. The results indicated a significant performance gap, with ETR consistently yielding smaller deviations compared to KNN. As depicted in Figure 4, the ETR predictions closely align with the line of best fit, unlike those from the KNN model. The mean absolute error (MAE) for PVmicro, EC, and FR using ETR ranged from 0.00 to 0.78, with the largest deviation observed for FR. In contrast, the KNN method exhibited larger absolute error deviations, up to 2.73 for FR, with the smallest being 0.02 for PVmicro. A similar trend was observed for case D2, where ETR consistently outperformed KNN. This superior performance of ETR can be attributed to its inherent capability for nonlinear regression, which iteratively models each feature with a regressor, using the other features as predictors, as opposed to the KNN method that relies on the values of its *k*-nearest neighbors determined by a distance metric.

**Figure 5:**
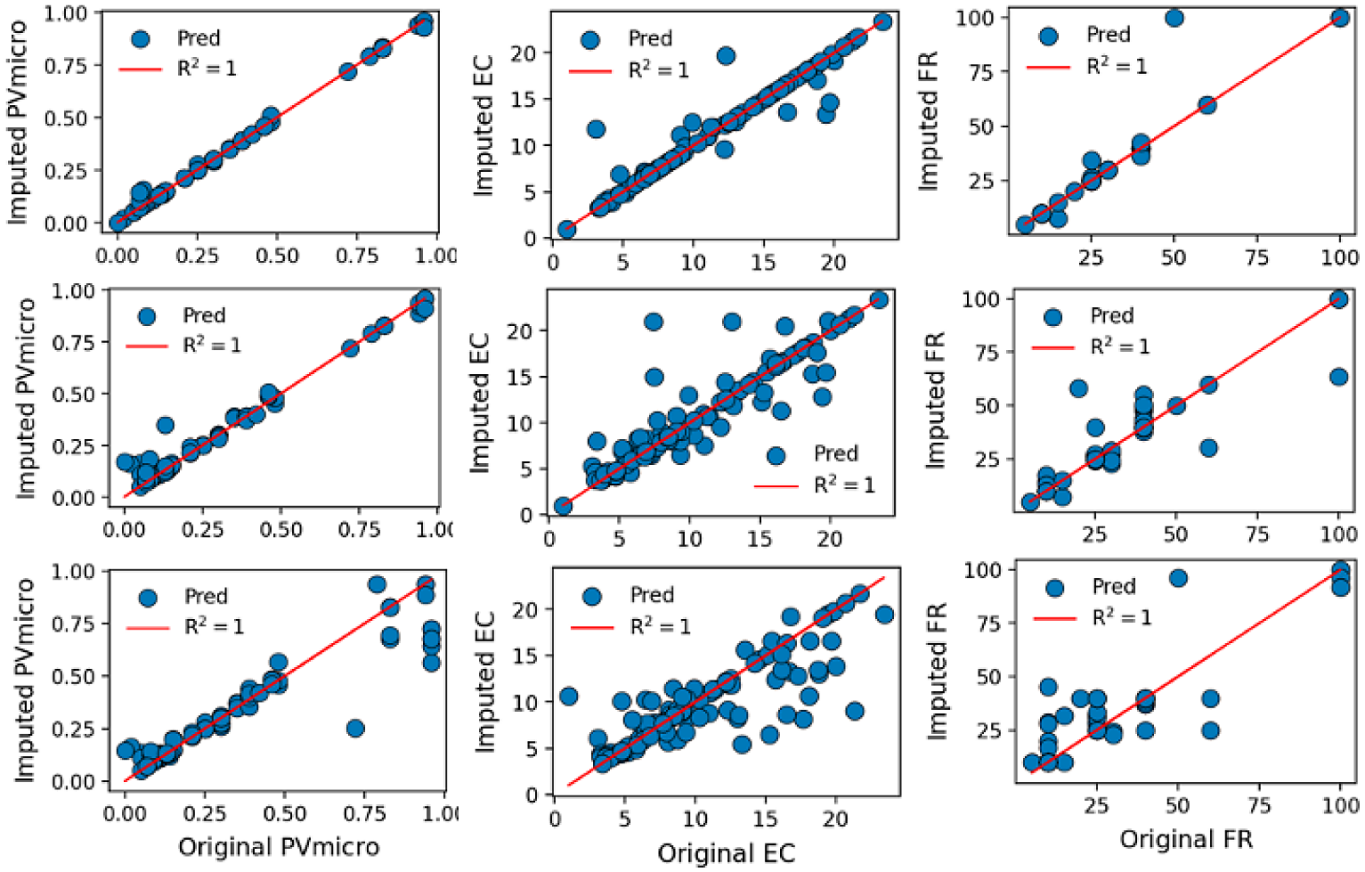
Effectiveness of data imputation with an increasing number of missing data for case D1. Top (20%), middle (50%), and bottom (70%).

Furthermore, we delved into the capacity of the best performing imputation model “ETR” to replicate databases characterized by substantial missing information. Typically, the percentage of missing data within a column could be as low as 20% to as high as 70%. To systematically assess the imputation model’s performance under such conditions, a missing database was meticulously constructed, incorporating varying percentages of missing data, namely 20%, 50%, and 70%. The ensuing comparison between ETR predictions and experimental data at different missing data percentages, as illustrated in Figures 5 and S2, revealed an unsurprising reduction in the model performance with increasing percentages of missing data. As detailed in Table 2, the MAE values for property FR at 20%, 50%, and 70% missing data were recorded as 0.78, 2.24, and 4.07, respectively. Analogous observations were made for other properties within cases D1 and D2 (in Table S2). The observed diminishing predictive accuracy underscores the intrinsic challenges associated with generating reliable imputations in the presence of extensive missing data. Although the imputation process offers commendable data generation capabilities, the resultant dataset falls short of aligning with the precision achieved through experimental measurements. Consequently, it is discerned that while imputation can furnish a viable dataset for pretraining the ML model, it may not fully replicate the intricacies inherent in experimental datasets. This nuanced understanding is crucial for practitioners seeking to leverage imputed data in ML model development, recognizing both its utility and limitations in approximating experimental precision.

**Table 2:** Performance of Imputation Models with the Increasing Count of Missing Data for Case D1.

	KNN			ETR		
% missing	PVmicro	EC	FR	PVmicro	EC	FR
20%	RMSE: 0.05 MAE: 0.02 *R*^2^: 0.97	RMSE: 1.68 MAE: 0.53 *R*^2^: 0.90	RMSE: 8.56 MAE: 2.73 *R*^2^: 0.81	RMSE: 0.01 MAE: 0.00 *R*^2^: 1.00	RMSE: 1.48 MAE: 0.45 *R*^2^: 0.92	RMSE: 5.05 MAE: 0.78 *R*^2^: 0.93
50%	RMSE: 0.08 MAE: 0.05 *R*^2^: 0.91	RMSE: 2.54 MAE: 1.28 *R*^2^: 0.76	RMSE: 8.30 MAE: 4.07 *R*^2^: 0.82	RMSE: 0.04 MAE: 0.01 *R*^2^: 0.98	RMSE: 2.28 MAE: 1.08 *R*^2^: 0.81	RMSE: 6.72 MAE: 2.24 *R*^2^: 0.88
70%	RMSE: 0.11 MAE: 0.06 *R*^2^: 0.85	RMSE: 2.95 MAE: 1.91 *R*^2^: 0.68	RMSE: 14.10 MAE: 7.62 *R*^2^: 0.49	RMSE: 0.09 MAE: 0.04 *R*^2^: 0.88	RMSE: 3.02 MAE: 1.69 *R*^2^: 0.67	RMSE: 8.99 MAE: 4.07 *R*^2^: 0.79

**Figure 6:**
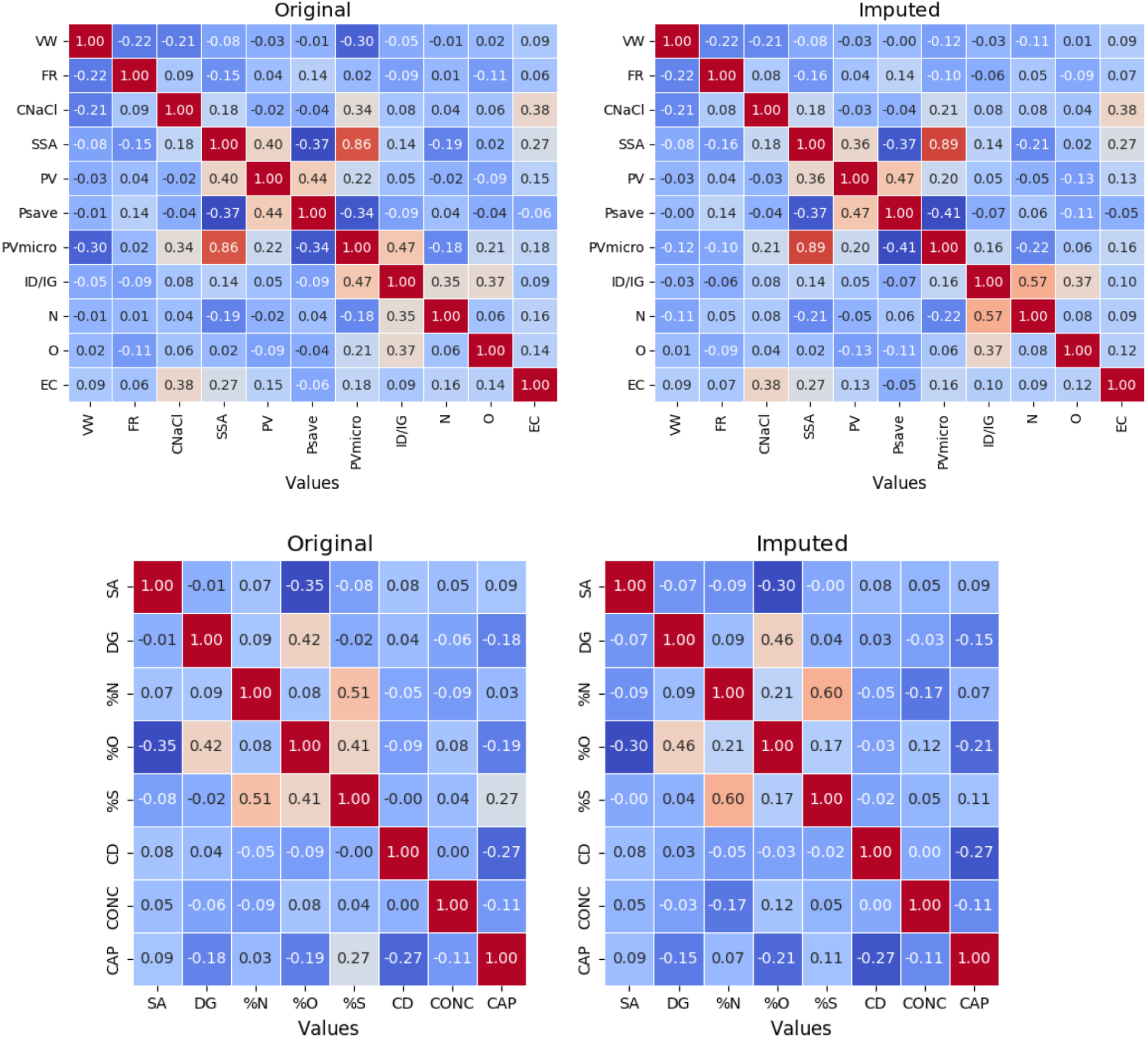
Comparison of the feature importance for the original dataset and imputed data for cases D1 (top) and D2 (bottom).

Having established the viability of imputation as a reliable data source, we proceeded to assess the preservation of relationships within datasets by generating a correlation map, as depicted in Figure 6. This correlation heatmap, derived from the Spearman correlation coefficient, elucidates the interplay among variables in both cases D1 and D2. Notably, the comparison involves the “original” which represents the experimental data with missing data and the “imputed” is the experimental data whose missing data has been populated with the selected ETR algorithm. To maintain the existing relationships, the missing dataset was intentionally retained within the original dataset, preventing the loss of crucial associations. The figures unveiled the sustained relationships between the datasets, exemplified by case D1, where EC correlates positively with selected features excluding Psave. Specifically, EC exhibits correlations of 0.09 and 0.16 with VW and N, respectively, in both the original and imputed datasets. Furthermore, EC shows correlations of 0.15 and 0.13 with PV in the original and imputed datasets, respectively. In case D2, a positive correlation was observed between CAP and SA, %N, and %S, and negative correlations were observed with DG, %O, CD, and CONC. The computed correlations for CAP and SA, CAP and CD, and CAP and CONC were 0.09, −0.27, and −0.11, respectively. While slight variations were noted for variables like DG, %N, and %O, these comparisons underscore the imputed dataset’s effective preservation of the overarching relationships within the original data, albeit with slight variation. In conclusion, the imputed dataset emerges as a commendable approximation of the original dataset, maintaining comparable central tendencies while displaying marginal variability.

### 3.2 Performance of the Machine Learning Model

**Figure 7:**
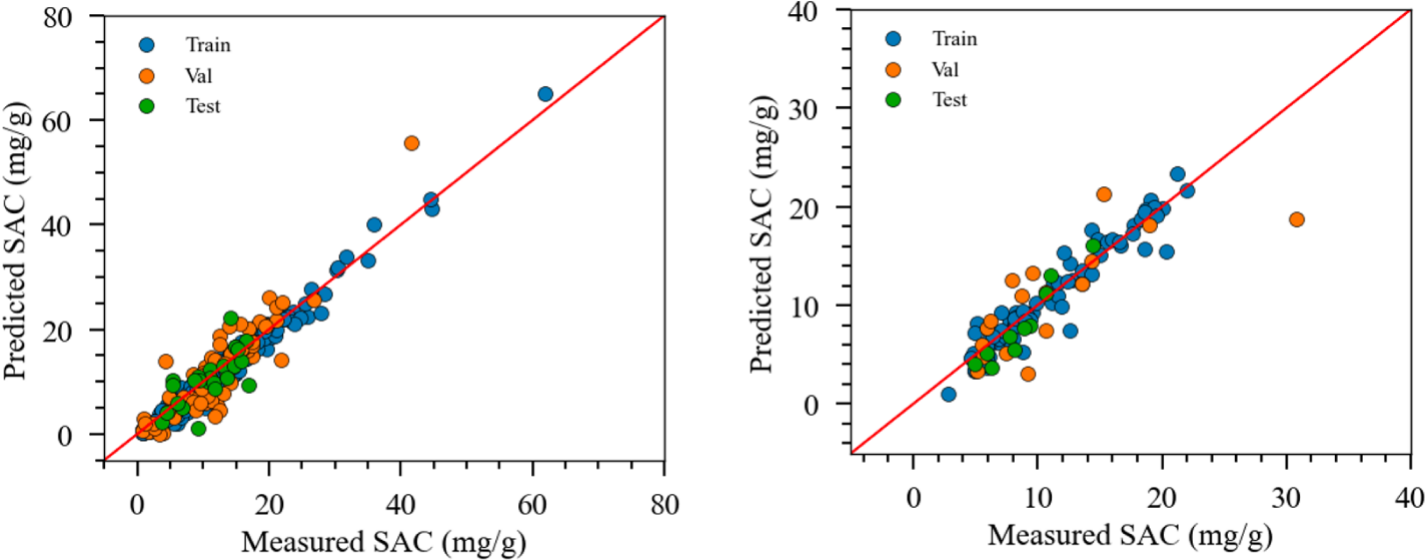
Cross-plot of the experimental vs model prediction of case D1 (salt adsorption capacity, mg/g). Left: source model and right: target model.

**Figure 8:**
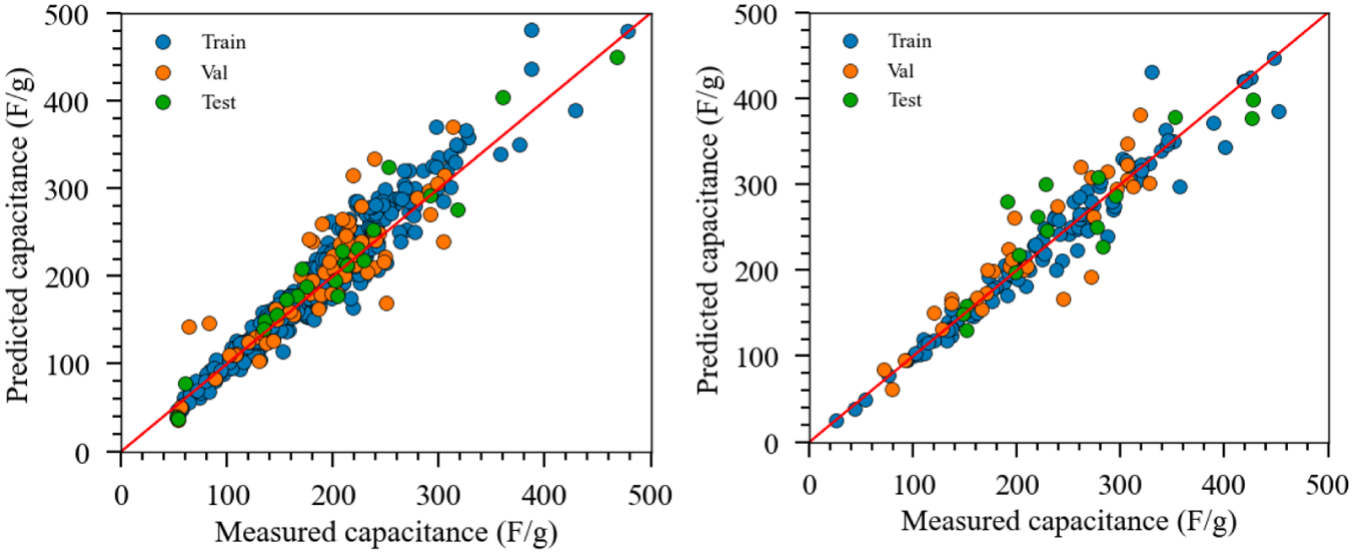
Cross-plot of the experimental vs model prediction of case D2 (capacitance, F/g). Left: source model and right: target model.

The initial phase of the proposed Transfer Learning (TL) methodology involves pretraining a neural network using imputed data, as described in Sections 2.3 and 3.1. In the model development process, an 80%:15%:5% random split into train, validation, and test sets was employed. To determine the optimal NN architecture for the source model, hyperparameter optimization was initiated using the Bayesian framework, yielding mean squared error values of 23.91 mg^2^/g^2^ and 3059.82 F^2^/g^2^ for cases D1 and D2, respectively. The pretraining dataset consisted of only the imputed data obtained through ML imputation (detailed in Section 3.1). Following feature scaling to a range between −1 and 1, the best NN architecture (as discussed in Section 2.3) for case D1 was identified as having five hidden layers, each with 50 nodes (12:50:50:50:50:1) and a learning rate of 0.001. For case D2, the optimal architecture comprised 10 hidden layers (12:21:38:9:35:45:50:14:50:10:1) and a learning rate of 0.001. During model training, the early stopping criterion with a mean squared error objective function and a patience of 40 ensured that the model halted if no improvement occurred. The resultant RMSE values (shown in Table 3) were notably low, with training, testing, and validation values of 1.26, 3.79, and 3.39 mg/g, respectively. Correspondingly, the mean absolute error (MAE) values were 0.94, 2.77, and 2.42 mg/g. Figures 7 and 8 visually demonstrate the alignment between the model predictions and the actual (imputed) data, indicating the model’s strong predictive accuracy and providing a robust foundation for the TL strategy. In case D2, similar high accuracy was observed, with MAE and RMSE values for training, validation, and the test sets.

**Table 3:** Performance Metrics of the Source and Target Model for Cases D1 and D2.

		source model		target model	
case	dataset	RMSE	MAE	RMSE	MAE
D1	train	1.26	0.94	1.49	1.04
	Val	3.79	2.77	4.13	2.96
	test	3.39	2.42	1.62	1.46
D2	train	20.98	15.40	21.14	12.29
	Val	50.47	27.96	33.57	25.63
	test	24.71	18.52	39.44	30.76

To construct the target model, the original dataset (sans missing data) served as the training dataset. Features were scaled and fed into the pretrained source model for fine-tuning. Despite a smaller dataset for training (<160 samples), the target model exhibited high prediction accuracy, with averaged RMSE values of 1.49, 4.13, and 1.62 F/g for the train (70%), validation (20%), and test (10%) sets. Importantly, this performance matched that of the source model, affirming successful knowledge transfer via the TL strategy without overfitting. Figures 7 and 8 further illustrate that most dataset points fall closely along the MAE equals 0 line, indicating nearly perfect predictions. This observation persisted in case D2, reinforcing the effectiveness of the TL scheme. Moreover, the TL approach can be enhanced by incorporating synthetic datasets, such as those obtained through imputation, to eliminate low and nonpredictive models, especially valuable when dealing with large datasets featuring missing values that might otherwise hinder meaningful model development.

### 3.3 Knowledge Extraction from the ML Model

**Figure 9:**
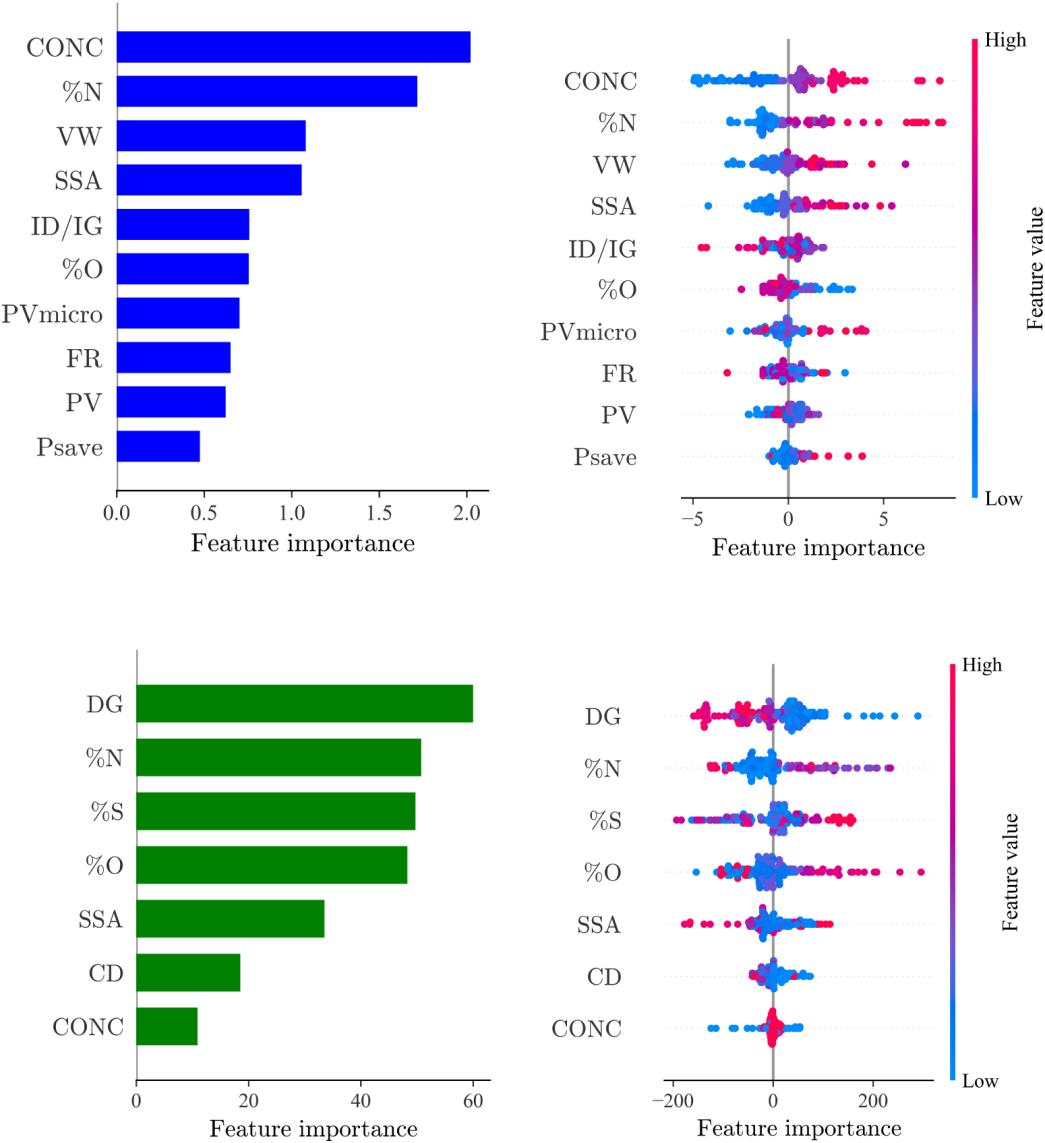
Feature importance characterized by SHAP values for the salt adsorption capacity (top) and capacitance (bottom) ML models.

Utilizing the TL-based NN model for predicting salt adsorption capacity (case D1) and specific capacitance (case D2), we delved into exploring the intricate relationship between input parameters and model outputs. By computing SHAP (SHapley Additive exPlanations) values from a dataset extracted from the “original data” parameter space, we derived importance scores and constructed SHAP summary plots, as illustrated in Figure 9. For each feature, a SHAP value is calculated, with positive values indicating an increase in the likelihood of a higher prediction outcome and negative values indicating a decrease. These plots provided a visual depiction of the influence of individual inputs on model predictions, offering valuable insights into the dynamics driving the predictive outcomes.

Based on the available training data, one standout observation was the pivotal role played by the electrolyte concentration (‘CONC’) in determining salt adsorption capacity. Notably, a cluster of mostly positive impact values surrounding ‘CONC’ indicated a correlation between elevated concentrations and increased salt adsorption capacity. Concurrently, features such as nitrogen content (‘%N’) and applied voltage window (‘VW’) displayed nuanced impacts, with a predominance of positive influences, showing their consistent association with higher predictive outcomes. Delving further into the nuanced dynamics of feature importance, the top 10 feature importance chart, elucidated by mean absolute SHAP values, revealed ‘CONC’ as the paramount influencer, closely followed by ‘%N’, ‘VW’, and ‘SSA’. The presence of typical heteroatoms, including nitrogen, oxygen, and sulfur, within the top 6 descriptors underscored their noteworthy influence on salt adsorption capacity. This comprehensive analysis not only sheds light on individual features but also provides a holistic understanding of their collective impact on the model’s predictive outcomes.

Transitioning to the specific capacitance task (case D2), a unique set of influential features emerged. The defect ratio and heteroatom content (%N, %O, and %S) took precedence as the top four most important features, with the electrolyte condition (”CONC”) exhibiting the least effect. These findings present a compelling narrative for electrochemists, offering actionable insights into the design of materials and process conditions to optimize electrochemical properties. By leveraging SHAP analysis, our study not only contributes to the fundamental understanding of the intricate relationships between input parameters and predictions but also empowers researchers in the pursuit of enhanced energy-efficient separations and generation through capacitive-based devices.

**Figure 10:**
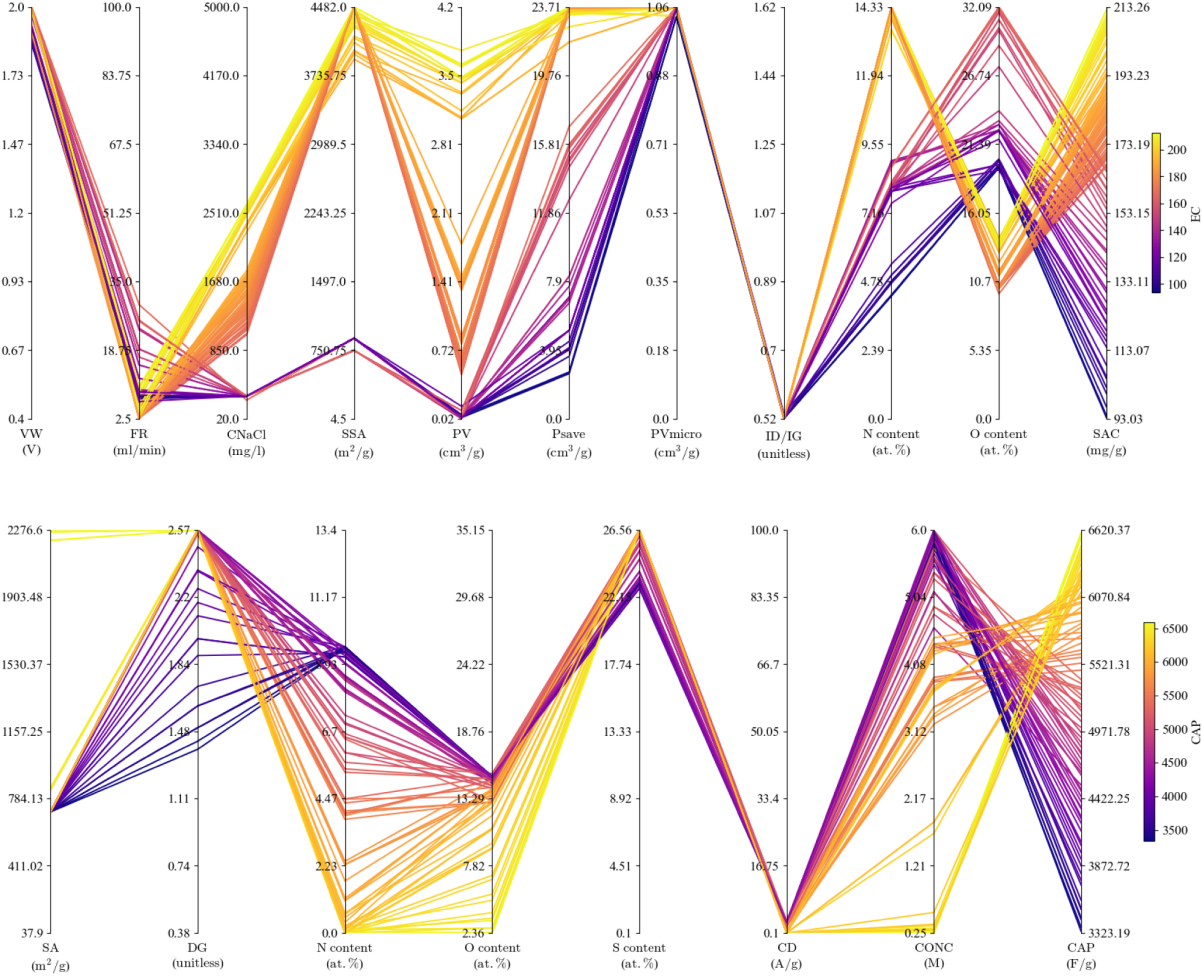
Solution in the Pareto optimality of the optimization problem for the salt adsorption capacity (top) and capacitance (bottom) ML models.

In an effort to delve deeper into the factors influencing optimal adsorption capacity or specific capacitance, we constructed a comprehensive optimization framework for the transfer learning (TL) model, following the approach outlined in ref^49^ and^50^. The Pareto front and the corresponding solutions of the optimization problem, as defined in eq 1, are thoughtfully presented in Figures 10 and S6. Our results show that the optimal performance for adsorption capacity and specific capacitance stands at impressive values of 200 mg/g and 6000 F/g, respectively. Remarkably, these values surpass the maximum reported experimental data by 3-fold for adsorption capacity and 10-fold for specific capacitance. Such heightened performance metrics, as revealed through our optimization framework, serve as invaluable benchmarks for experimental pursuits, guiding researchers toward candidate solutions that exhibit superior electrochemical properties. In conclusion, our findings not only contribute to the understanding of optimal conditions through data-driven optimization but also offer practical insights into the experimental exploration of capacitive devices, ultimately advancing the landscape of energy-efficient separation and generation.

## 4 Conclusion

In addressing the challenge posed by missing data in capacitive devices, our research introduces a comprehensive approach that combines data imputation with neural network machine learning models. This innovative strategy enables the modeling and prediction of critical parameters such as salt adsorption capacity (SAC) in capacitive deionization and specific capacitance (CAP) in supercapacitors spanning diverse operational and material conditions. Leveraging a transfer learning (TL) scheme, we employed imputed data—filled through machine learning—to initially train a model and subsequently refine it with clean data devoid of missing values. The success of the optimized TL-based neural network model underscores the efficacy of the pretraining scheme. Feature analysis outcomes underscored the predominant impact of electrolyte concentration on deionization SAC and the significance of the defect ratio on supercapacitor CAP. To explore optimal feature combinations, we employed the NSGA-II algorithm, a metaheuristic approach, generating potential experimental conditions within the current design space. Our algorithm, designed with meticulous examination over model parameters and dataset accuracies, yielded a parsimonious yet highly accurate surrogate model in the mixed data regime. This research thus provides a robust framework for addressing missing data challenges, offering insights into influential factors, and paving the way for efficient exploration of experimental conditions in capacitive devices.

## Data Availability

The coding available at https://github.com/EnthusiasticTeslim/ImputeNet was conducted in Python 3, utilizing TensorFlow for the creation of neural networks and Scikit-learn for the KNN imputer, MICE imputer, and Extra Tree regressor. Additionally, for data handling and visualization with Pandas, Seaborn, Matplotlib, and NumPy.

## Supporting Information Available

Detailed information on the statistical analysis and model performance for case D2 (specific capacitance), the SHAP dependence plot, the 2D projection for training data, and the multi-objective framework and the resulting pareto front (PDF)
