# Supplementary material for: Empowering Capacitive Devices: Harnessing Transfer Learning for Enhanced Data-Driven Optimization
Source: Ind Eng Chem Res. 2024 Jun 29;63(27):11971–81. doi: 10.1021/acs.iecr.4c01171 (PMC11247430; doi:10.1021/acs.iecr.4c01171)
Supplement: Supplementary file 1 — ie4c01171_si_001.pdf [file ie4c01171_si_001.pdf]

Empowering Capacitive Devices: Harnessing Transfer Learning for Enhanced Data-Driven Optimization

Teslim Olayiwola[1], Revati Kumar[2], Jose A. Romagnoli[1,*]

[1]*Cain Department of Chemical Engineering, Louisiana State University, Baton Rouge, Louisiana 70803, United States.*

[2]*Department of Chemistry, Louisiana State University, Baton Rouge, Louisiana 70803, United States.*

[*]Corresponding authors: jose@lsu.edu

Table S1. Summary of statistics analysis of the *case D2*.

	SA	DG	%N	%O	%S	CD	CONC	CAP
count	620	541	402	542	236	620	620	620
mean	580.42	1.14	4.02	12.28967	2.26	6.25	5.17	204.74
std	575.59	0.33	2.99	6.494863	3.93	9.18	1.87	81.51
min	37.9	0.38	0	2.36	0.1	0.1	0.25	25
25%	196.8	0.98	1.52	7.2725	0.7	1	6	150
50%	350.9	1.05	2.97	11.2	1.2	3	6	200
75%	710	1.28	5.88	15.45	2.39	10	6	253.4
max	2276.6	2.57	13.4	35.15	26.56	100	6	566

Table S2. Performance of imputation models with increasing count of missing data for *case D2*.

% missing	KNN			EXT		
	%N	SA	DG	%N	SA	DG
20%	RMSE: 0.70<br>MAE: 0.19<br>R^2: 0.92	RMSE: 166.05<br>MAE: 48.36<br>R^2: 0.87	RMSE: 0.08<br>MAE: 0.02<br>R^2: 0.88	RMSE: 0.07<br>MAE: 0.01<br>R^2: 1.00	RMSE: 9.92<br>MAE: 1.74<br>R^2: 1.00	RMSE: 0.02<br>MAE: 0.00<br>R^2: 0.99
50%	RMSE: 1.02<br>MAE: 0.49<br>R^2: 0.84	RMSE: 381.24<br>MAE: 161.44<br>R^2: 0.30	RMSE: 0.12<br>MAE: 0.06<br>R^2: 0.70	RMSE: 0.27<br>MAE: 0.08<br>R^2: 0.99	RMSE: 62.70<br>MAE: 19.83<br>R^2: 0.98	RMSE: 0.04<br>MAE: 0.02<br>R^2: 0.96
70%	RMSE: 1.37<br>MAE: 0.78<br>R^2: 0.71	RMSE: 383.66<br>MAE: 230.64<br>R^2: 0.29	RMSE: 0.18<br>MAE: 0.10<br>R^2: 0.30	RMSE: 0.65<br>MAE: 0.33<br>R^2: 0.93	RMSE: 112.83<br>MAE: 43.59<br>R^2: 0.94	RMSE: 0.11<br>MAE: 0.05<br>R^2: 0.74

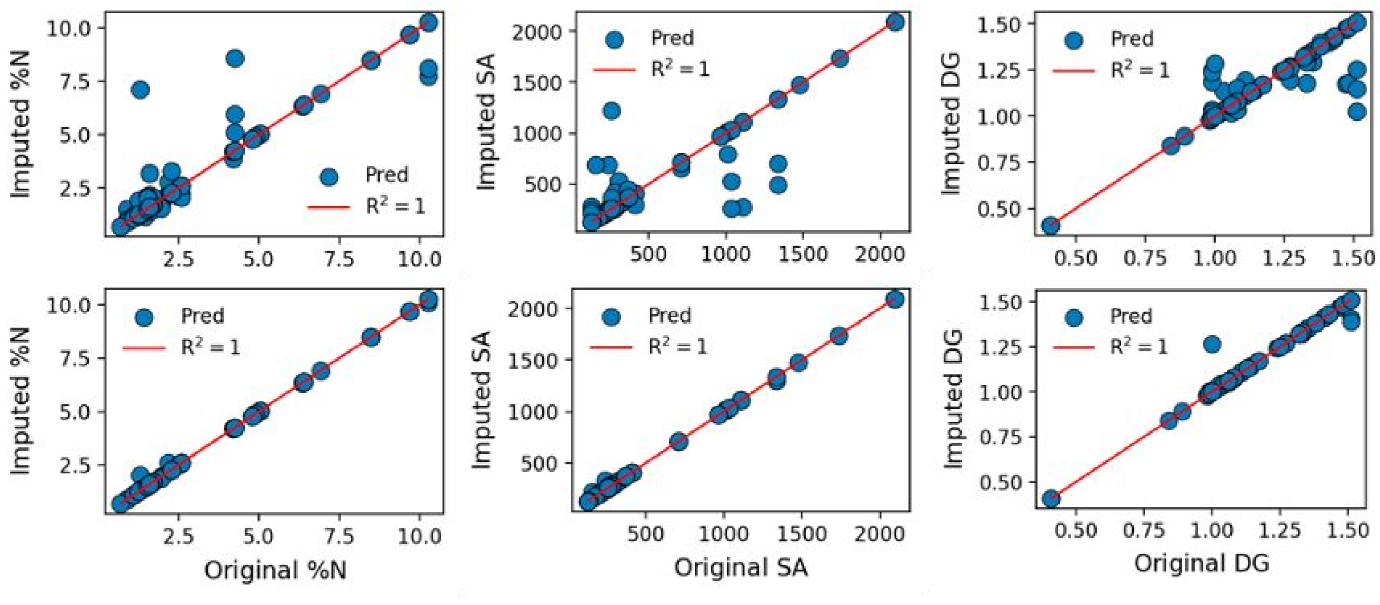

Figure S1. Effectiveness of the data imputation for *case D2* (Specific capacitance, F/g).

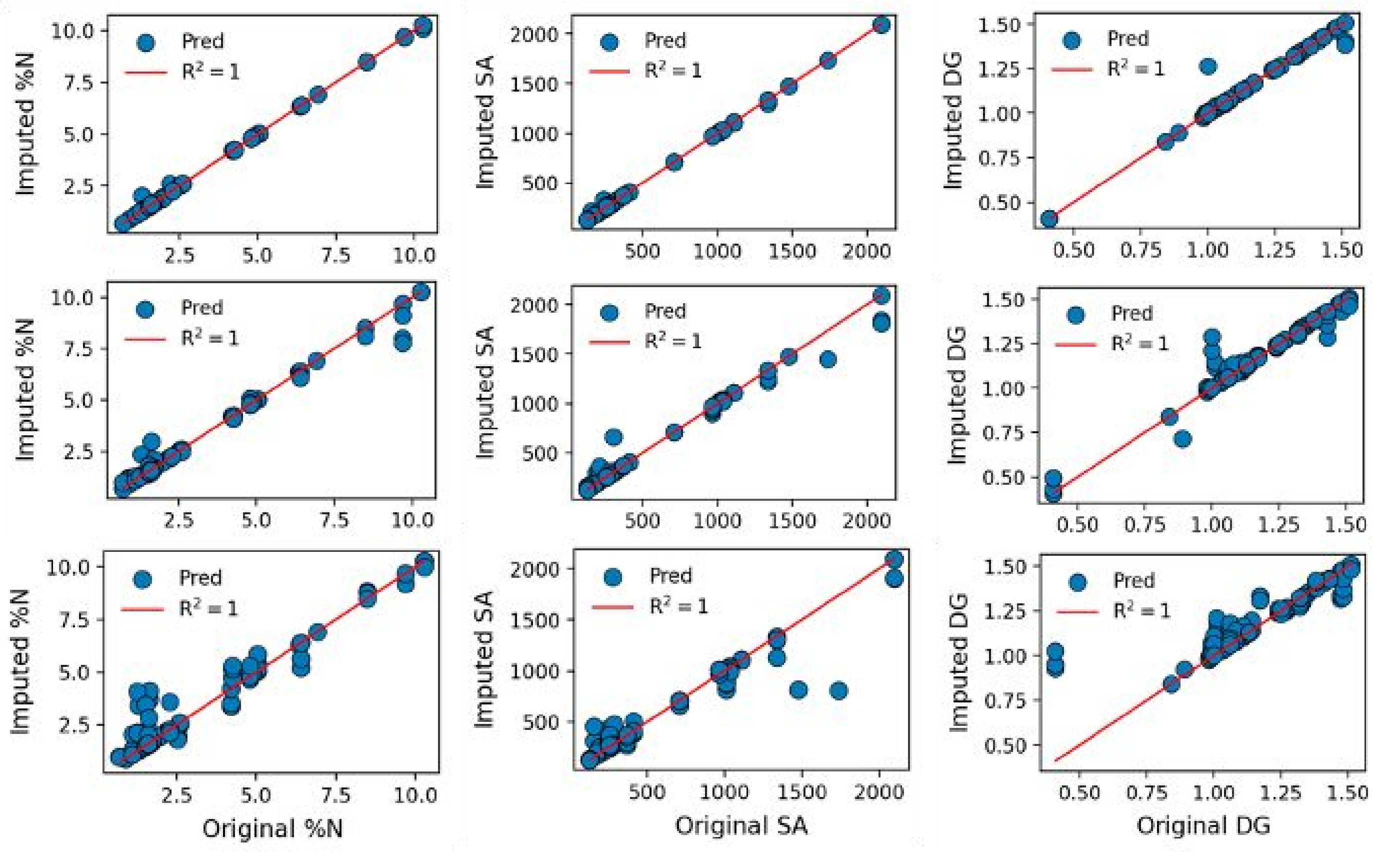

Figure S2. Effectiveness of the data imputation with increasing number of missing data for *case D2* (Specific capacitance, F/g).

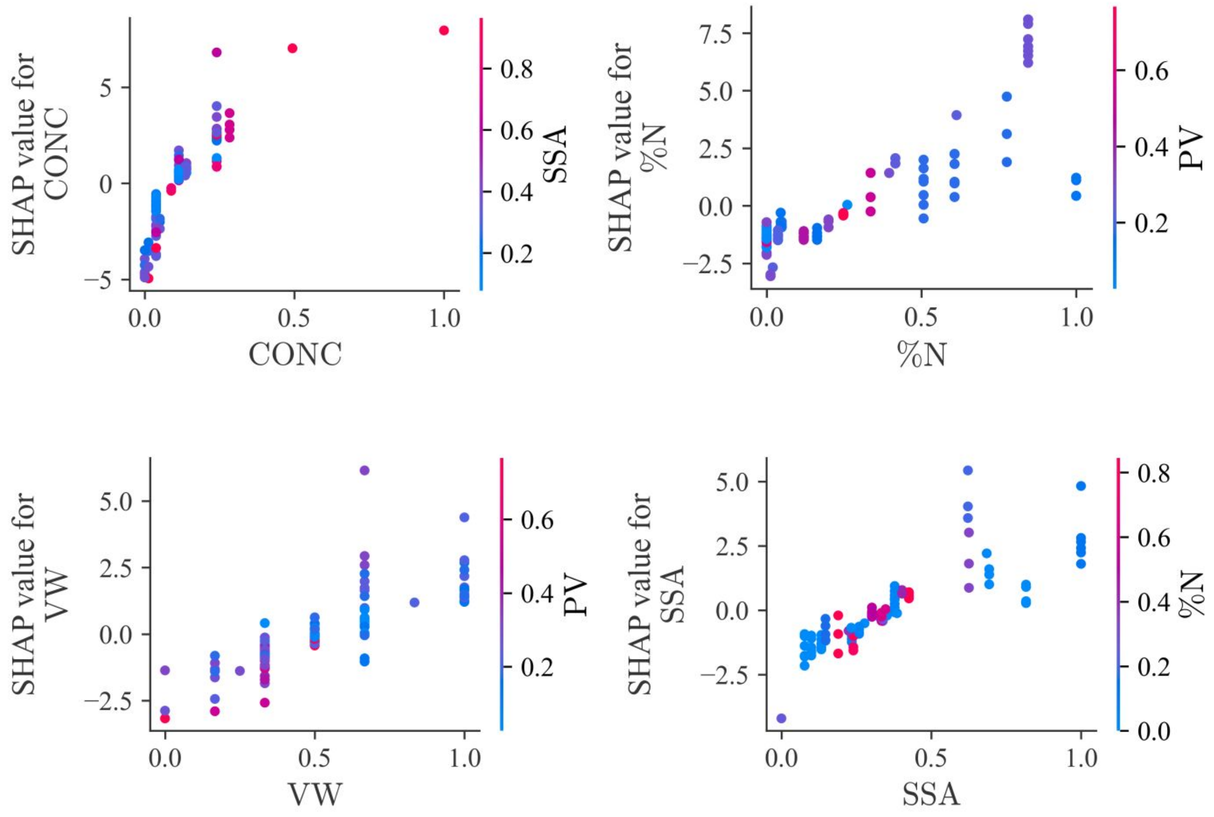

Figure S3: SHAP Dependence Plot for top 4 features in Case *D1* with SHAP values (on primary y-axis) indicating the contribution of each feature to the salt adsorption capacity (i.e. model prediction). The color bar represents the interaction level with another randomly feature in the input list.

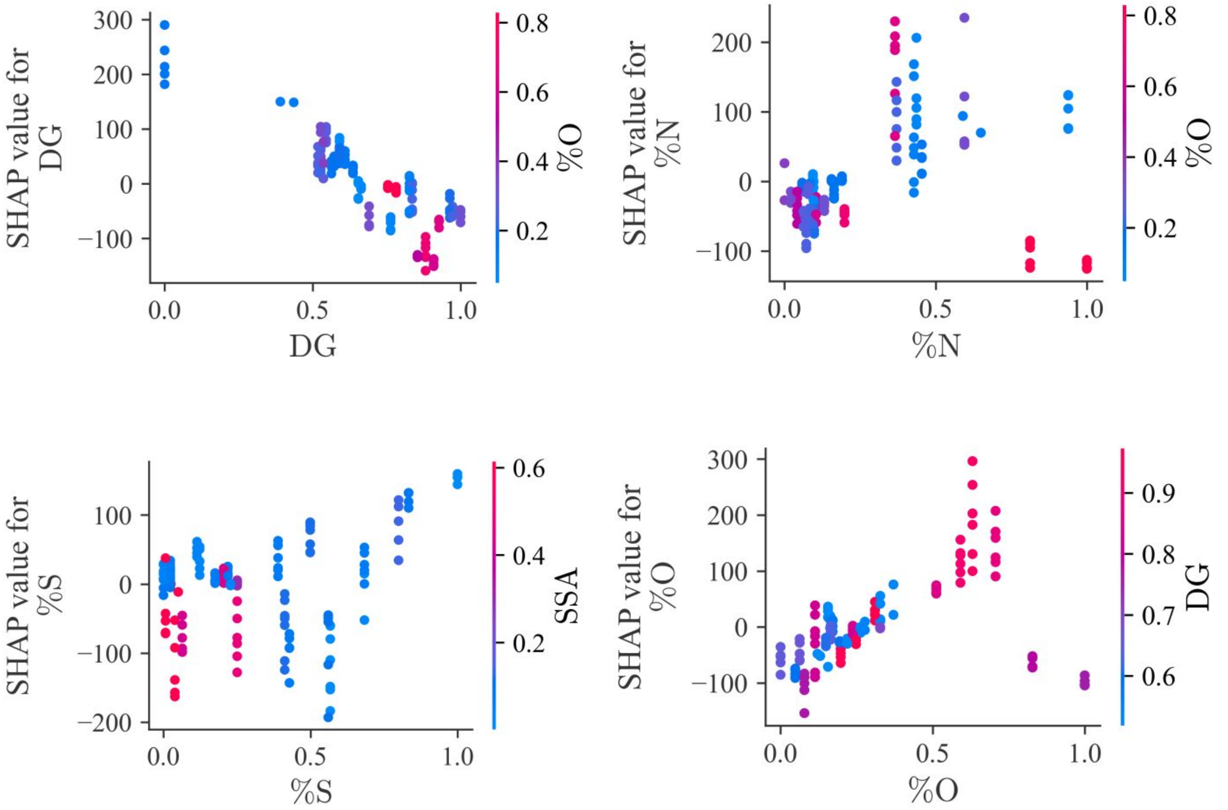

Figure S4: SHAP Dependence Plot for top 4 features (x axis) in Case *D2* with SHAP values (y-axis) indicating the contribution of each feature to the *specific capacitance* (i.e. model prediction). The color bar represents the interaction level with another randomly feature in the input list.

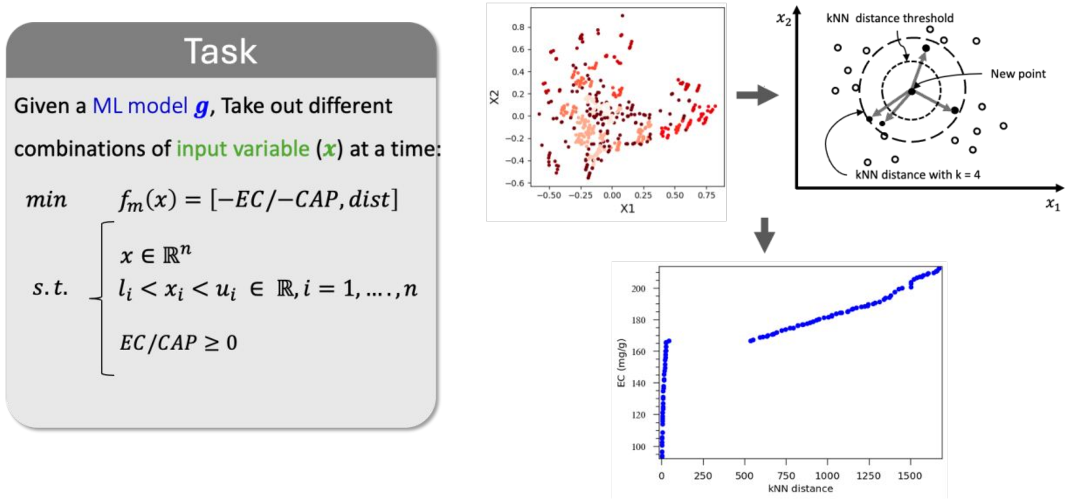

Figure S5. Multi-objective Optimization employed in this study

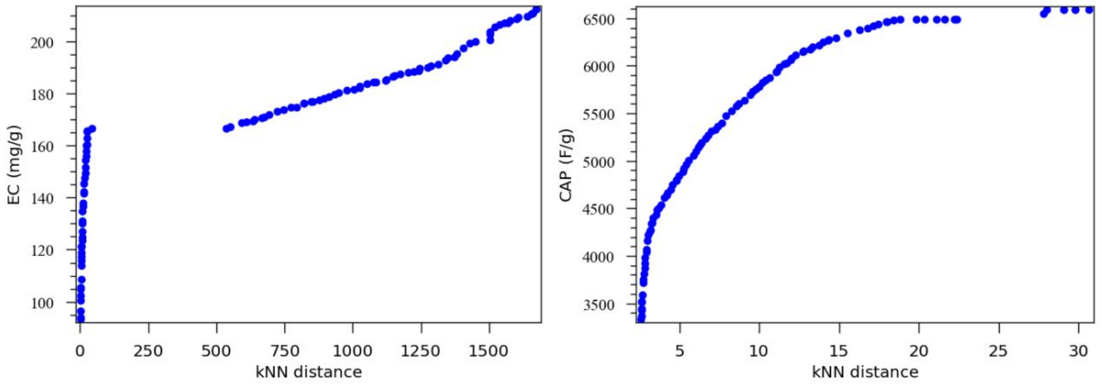

Figure S6. Pareto front for multi-objective optimization problem defined in eq. (1). Left (case *D1*, Salt adsorption capacity, mg/g); Right (case D2, Specific capacitance, F/g). The KNN threshold for case D1 and D2 is 1500 (based on 3 KNN neighbors) and 70.41 (based on 5 KNN neighbors), respectively.

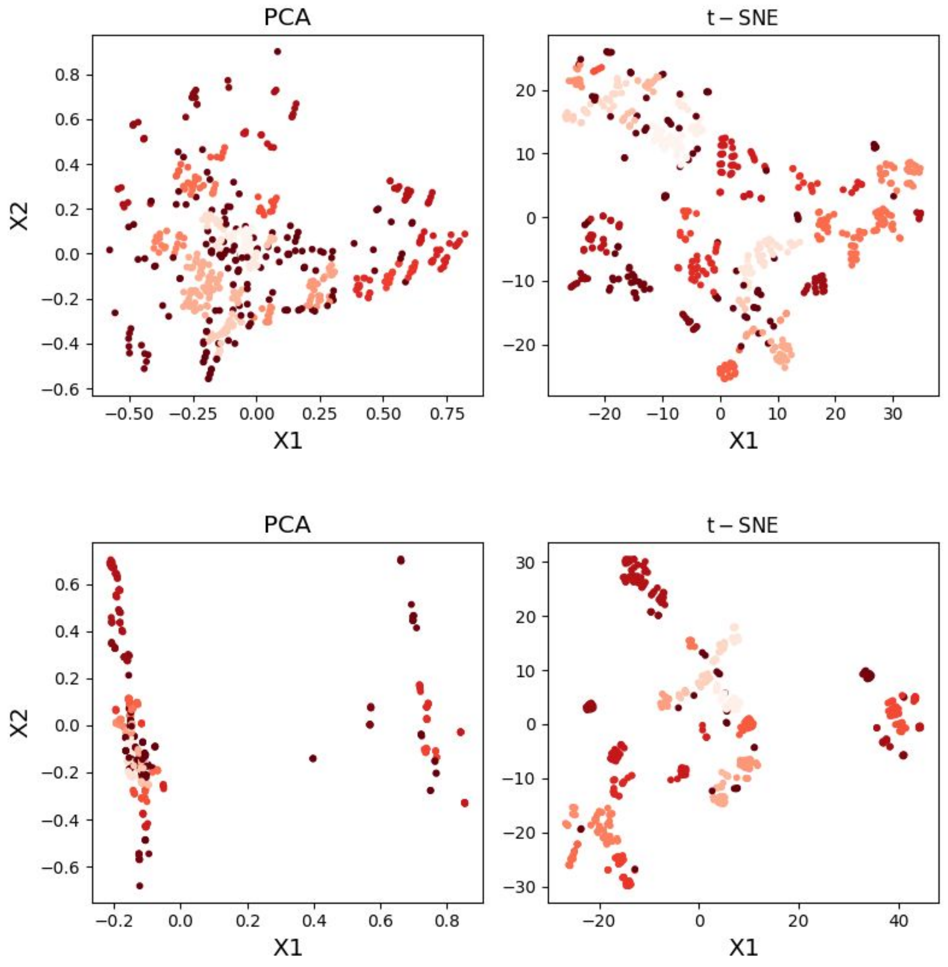

Figure S7. 2D projection for training data via PCA and tSNE dimensionality reduction algorithm. Top (case *D1*, Salt adsorption capacity, mg/g); Bottom (case D2, Specific capacitance, F/g).
